# Strain Engineering of Germanium Nanobeams by Electrostatic Actuation

**DOI:** 10.1038/s41598-019-41097-1

**Published:** 2019-03-21

**Authors:** Arman Ayan, Deniz Turkay, Buse Unlu, Parisa Naghinazhadahmadi, Samad Nadimi Bavil Oliaei, Cicek Boztug, Selcuk Yerci

**Affiliations:** 1 Department of Electrical and Electronics Engineering, Middle East Technical University, Ankara, 06800 Turkey; 2 Center for Solar Energy Research and Applications, Middle East Technical University, Ankara, 06800 Turkey; 3 Department of Micro and Nanotechnology, Middle East Technical University, Ankara, 06800 Turkey; 4 Department of Electrical and Electronics Engineering, TED University, 06420 Ankara, Turkey; 5 Department of Mechanical Engineering, Atilim University, 06836 Ankara, Turkey

## Abstract

Germanium (Ge) is a promising material for the development of a light source compatible with the silicon microfabrication technology, even though it is an indirect-bandgap material in its bulk form. Among various techniques suggested to boost the light emission efficiency of Ge, the strain induction is capable of providing the wavelength tunability if the strain is applied via an external force. Here, we introduce a method to control the amount of the axial strain, and therefore the emission wavelength, on a suspended Ge nanobeam by an applied voltage. We demonstrate, based on mechanical and electrical simulations, that axial strains over 4% can be achieved without experiencing any mechanical and/or electrical failure. We also show that the non-uniform strain distribution on the Ge nanobeam as a result of the applied voltage enhances light emission over 6 folds as compared to a Ge nanobeam with a uniform strain distribution. We anticipate that electrostatic actuation of Ge nanobeams provides a suitable platform for the realization of the on-chip tunable-wavelength infrared light sources that can be monolithically integrated on Si chips.

## Introduction

Monolithic integration of electronics and photonics on the same chip would enable breakthrough advances in infrared technologies serving to a variety of fields ranging from biochemical sensing to optical communications. More specifically, such integration could make it possible to develop miniaturized, low-cost and, therefore, potentially disposable lab-on-a-chip biosensors if the photonic components are designed to operate at mid-infrared (MIR) wavelengths where most of the biochemical species have distinct absorption features. Furthermore, the integration of microelectronics with photonics operating at 1550 nm would provide chip-level optical communication with superior data transfer rates. The main obstacle to realize such fully-integrated systems is the lack of efficient light emitters compatible with the silicon (Si) microfabrication technology.

Germanium (Ge) has been attracting significant attention of researchers for around a decade due to its potential to be converted into an efficient CMOS-compatible light source despite its inherent indirect-bandgap nature^1–8^. The techniques utilized to enhance the light emission efficiency of Ge include heavy n-type doping^9–11^, tin (Sn) incorporation^12–16^ and introduction of tensile strain^5,10,17–32^ in Ge. n-type doping is generally accompanied by a small amount of tensile strain, where, strain reduces the 140-meV energy difference between the direct and indirect band gaps to some degree, and the remaining energy difference is compensated by the extrinsic electrons of dopants that fill the indirect valleys (L-valleys) of Ge. In fact, an electrically pumped Ge laser on Si has been demonstrated utilizing this technique. However, it suffers from the extremely high threshold current density (280 kA/cm^2^) mainly due to recombination losses associated with the required high doping concentrations^9^. Sn incorporation and high tensile strain induction are the two other techniques used to enhance the light emission efficiency of Ge by lowering direct-bandgap edge of the conduction band (Γ-valley) relative to the L-valleys, which in turn gives rise to direct bandgap Ge if Sn concentration or the amount of tensile strain is sufficiently high. For example, GeSn alloy with an Sn concentration of around 13% and Ge with an introduced biaxial strain of around 1.9% exhibit direct bandgap^13,33^. However, the low equilibrium solubility of Sn in Ge (~1%) makes it very challenging to realize GeSn with such high Sn concentrations^13–16^. On the other hand, application of tensile strain in Ge is a well-resolved technique employed by several research groups, leading to the demonstration of room-temperature light emission as well as the demonstration of direct-bandgap Ge. The strain induction methods include 1) heteroepitaxial growth of Ge on a buffer layer with larger lattice constant^34–38^ (such as InGaAs and GeSn), 2) strain transfer from a stressor layer (such as silicon nitride and tungsten) to Ge film^22,39,40^ or lithographically-patterned Ge nanostructures^19,41–43^, 3) application of external stress through a micromechanical module^44^ or the injection of high pressure gas^18,26,29,30^ and 4) redistribution of the intrinsically-introduced strain in Ge-on-Si to the suspended Ge microstructures^17,20,27,28,45–47^. Among these techniques, the application of an external force provides an additional advantage, namely the strain-tunability. For example, Greil *et al*.^44^ and Sanchez-Perez *et al*.^26^ tuned the strain in a controllable fashion by varying the magnitude of the applied force. The strain tunability could be utilized to demonstrate tunable-wavelength on-chip Ge lasers if the external force is applied in a form compatible with the integrated circuit (IC) technology.

In this study, we introduce a technique that is capable of inducing and tuning the tensile strain in Ge through the application of an electrostatic force, which is perfectly compatible with the IC technology. The electrostatic force is exerted on a suspended Ge nanobeam in a simulation domain by means of a voltage applied between the terminals of the electrically-insulated Ge nanobeam and Si substrate. The Ge nanobeam deflects under the effect of this force, and the deflection induces tensile strain in the Ge nanobeam. The finite element method (FEM) simulations demonstrate that an axial tensile strain above 4% can be achieved, and the required voltage to achieve a desired axial strain can be reduced using a stressor layer. Finally, we present the results of our electrical simulations, where we demonstrate that the graded band gap of deflected nanobeams enhances light emission compared to uniformly-strained nanobeams.

## Results and Discussion

### Design of the electrostatic strain induction on Ge nanobeams

**Figure 1:**
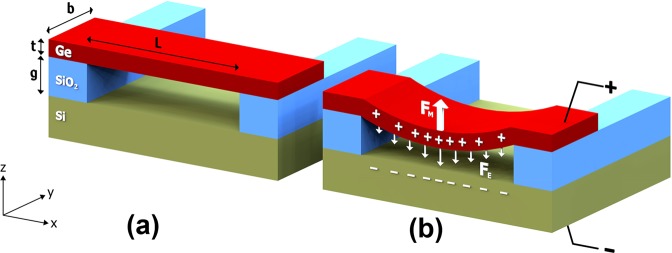
Cross-sectional schematic of the Ge nanobeam structure. Ge nanobeam suspended from both sides by SiO_2_, (**a**) before and (**b**) after applied voltage. L, b and t in (**a**) represent the length, width and thickness of the nanobeam, respectively. g in (**a**) indicates the gap between the Ge nanobeam and Si substrate. Plus and minus signs in (**b**) represent positive and negative charges accumulated at the nanobeam and substrate, respectively. Electrical and mechanical forces balancing the nanobeam are represented in (**b**) by downward and upward arrows, respectively.

A cross-sectional schematic of a prismatic suspended Ge nanobeam, supported by an insulator on its edges, is shown in Fig. 1a. When a potential difference is applied between the Si substrate and Ge nanobeam, positive and negative charges attract each other and an electrical force establishes, resulting in deflection of the nanobeam. Simultaneously, a mechanical force is formed by the interatomic interactions in the opposite direction. As a result, the deflected nanobeam remains in equilibrium under the effect of these two forces as depicted in Fig. 1b. The transverse displacement (i.e. deflection) of the nanobeam (w) exhibits a maximum at the midpoint between the two insulators, also referred to as the plane of symmetry.

**Figure 2:**
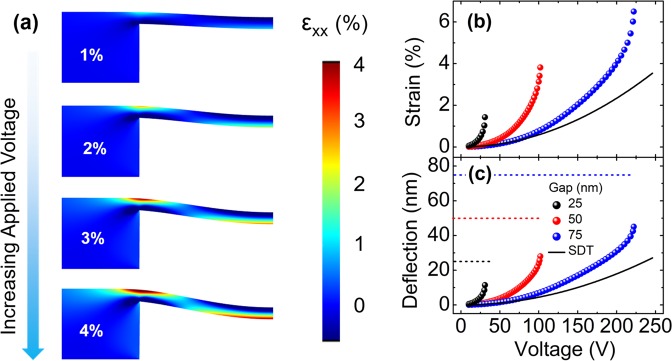
(**a**) Tensile strain profiles of Ge nanobeams for maximum axial strains of 1%, 2%, 3% and 4% on the bottom surface of the nanobeam at the plane of symmetry. The variation of strain (**b**) and deflection (**c**) with applied voltage for L = 350 nm, t = 20 nm and three different gap values of 25 nm (black spheres), 50 nm (red spheres) and 75 nm (blue spheres). Black dashed lines are the expected trends based on the small deflection theory (SDT) for a gap of 75 nm. Maximum strain and deflection values for various gaps in (**b**) and (**c**) are their maximum achievable values right before the pull-in occur.

As the nanobeam bends with the applied voltage, an axial strain (ε) is formed on it as shown in Fig. 2a and in the Supplementary Information, Video 1. Local maxima of the axial tensile strain occur on the bottom surface of the nanobeam at the plane of symmetry and on the top surface of the nanobeam at the two edges. In this study, we focused on the analysis of the axial tensile strain at the bottom surface of the nanobeam at the plane of symmetry, as the axial strain at the two edges depends significantly on the geometry of the corners as shown in Supplementary Fig. S1.

The variation of the axial strain and deflection on the bottom surface of the nanobeam at the plane of symmetry with the applied voltage (V) are shown in Fig. 2b,c, respectively. It should be noted that 2D electromechanical simulations provide an upper bound for the applied voltage as demonstrated in Supplementary Fig. S2 and as discussed in Supplementary Information, Section 1. The axial strain and deflection increase with the square of the applied voltage at relatively small deflections as predicted by the small deflection theory (Supplementary Information, Section 2). However, as the deflection is further increased, these trends deviate from the predictions indicating that small deflection theory is not valid anymore.

For large deflections, one needs to include geometric nonlinearities (Supplementary Information, Section 3) associated with the coupling between the transverse displacement (i.e. deflection) and the axial strain^48,49^. Additionally, the effects of the elasticity of the SiO_2_ on which the Ge nanobeam is resting (Supplementary Fig. S3) and the electrical force distribution on the nanobeam that changes as the nanobeam bends (Supplementary Fig. S4) should be included in the model.

As it is illustrated in Fig. 2b, the required voltages to reach sufficiently large strain values in Ge nanobeams are relatively large; nevertheless, strain induction in Ge via electrostatic force is a novel and viable technique if these voltage levels can be reduced down to the values typically used in on-chip applications. The required voltage to reach a predefined strain can be reduced by (1) narrowing the gap between the Si and Ge, (2) optimizing L and t of the nanobeam, and (3) inducing an initial strain on the nanobeam. Figure 2b shows that the required voltage to obtain the same strain (e.g. 2%) decrease significantly by reducing the gap between the two terminals. Yet, the maximum achievable strain also decreases with decreasing gap as shown in Fig. 2b.

As the ratio of the deflection to the gap exceeds a certain value, the electrical force overtakes the mechanical one. As a result, the nanobeam snaps to the substrate, which is known as the ‘pull-in’. Once the pull-in occurs, a large tensile strain is induced on the nanobeam; however, the nanobeam loses its strain-tunability feature^50^. Under the assumptions of a parallel plate capacitor, the stiffness being linear with the deflection and the neglected fringing fields, the deflection where the pull-in occurs is calculated as one third of the gap^50,51^. However, in various studies, a stable deflection well-exceeding one third of the gap (g/3) has been demonstrated^52,53^ and various methods have been offered to extend the range of operation^54–57^. Figure 2c demonstrates that deflections around g/2 can be obtained prior to pull-in in Ge nanobeams. However, a deflection equal to g/3 is used throughout the rest of this study to be able to achieve a desired strain while avoiding pull-in and benefiting from voltage-tunability of the strain.

**Figure 3:**
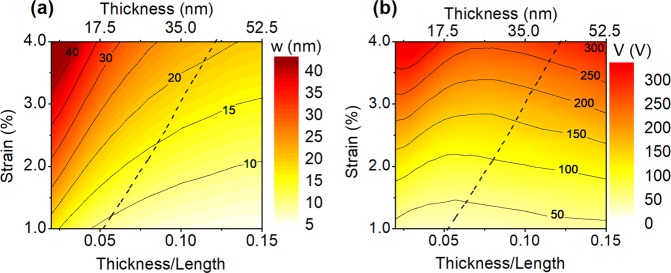
The deflection (**a**) and the applied voltage (**b**) for various axial strains and t/L ratios. The length of the nanobeam is set to 350 nm. The distance between the Ge nanobeam and Si substrate is equal to 3 times of the deflection shown in (**a**). Dashed line indicates w = t/2, and roughly represents the boundary between the large and small deflection conditions.

The deflection and the applied voltage for various axial strains and t/L ratios at a fixed L of 350 nm are shown in Fig. 3a,b, respectively. Small deflection theory (Supplementary Information, Section 2) can principally explain the trends in deflection and voltage with the axial strain for the data points appearing at the right side of the dashed line in Fig. 3a,b, where deflections are smaller than half of the nanobeam thickness^58^. This can further be confirmed when Fig. 3 is compared with the data presented in Supplementary Fig. S5, where non-linear effects are excluded in the simulation. As predicted by the small deflection theory, at large t/L values, the deflection increases linearly with the axial strain (Fig. 3a). Similarly, the required voltage to achieve a predetermined axial strain increases with ε^1.5^ as shown in Fig. 3b.

When the deflection becomes larger than the half of the nanobeam thickness (i.e. the left side of the dashed lines in Fig. 3a,b), the deflection and voltage variation with strain deviate from the ones predicted by the small deflection theory. In large deflections, for a constant t/L ratio, strain increases superlinearly with deflection mainly due to strain localization (Supplementary Information, Section 3). On the other hand, strain increases with V^a^ where a is slightly smaller than the value predicted by the small deflection theory (i.e. 2/3) for a constant t/L ratio mainly due to stress-stiffening (Supplementary Information, Section 3). In other words, the effect of the strain localization is suppressed by that of stress-stiffening. Relatively small Young’s modulus of SiO_2_ leads to an increase in the required deflection and voltage to achieve a predetermined strain. This is, in particular, obvious when Fig. 3 is compared with Supplementary Fig. S6a,b, which show the deflection and the applied voltage for various axial strains and t/L ratios for a fixed-fixed boundary condition, respectively.

The variation of the required voltage with t/L is rather intricate. The effect of stress-stiffening reduces as t/L increases. Thus, the applied voltage required to achieve a predetermined axial strain decreases with t/L ratio at large deflections as shown in Fig. 3b. A higher voltage needs to be applied with increasing t/L since the effect of shear deformation on nanobeam deflection increases (Supplementary Information, Section 3). As a result, at an optimum t/L ratio of around 0.07, the required applied voltage can be minimized. Additionally, the deflection and applied voltage vary linearly with the length of the Ge nanobeam for a fixed thickness as shown in Supplementary Fig. S7.

### Electrostatic strain induction on initially strained Ge nanobeams

**Figure 4:**
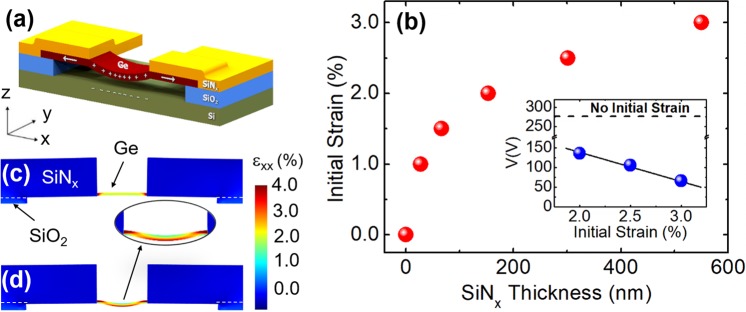
(**a**) Cross-sectional schematic of the Ge nanobeam, initially strained by a SiN_x_ layer, under the presence of an electrostatic force. The length, width and thickness of the Ge nanobeam is 350 nm, 218 nm and 20 nm, respectively. White arrows indicate tension introduced by SiN_x_. Plus and minus signs represent positive and negative charges accumulated at the nanobeam and substrate, respectively. (**b**) The variation of the initial strain with SiN_x_ thickness for a SiO_2_ thickness of 100 nm. SiN_x_ is assumed to have an initial tensile stress of 1.5 GPa. The separation between SiO_2_ at the both sides of the Ge nanobeam is 1400 nm. Inset: The required voltages to achieve 4% axial strain (on the bottom surface of the nanobeam at the plane of symmetry) for various initial strain values. The dashed line in the inset represents the required voltage to achieve 4% axial strain without any initial strain. (**c**) Tensile strain profile of the 2%-strained Ge nanobeam by a SiN_x_ stressor. (**d**) Tensile strain profile of the Ge nanobeam in (b) upon the application of 136 V to achieve 4% axial strain. Inset of (d) is a magnified image of the strain profile on the Ge nanobeam shown in (d). The gap between the Ge nanobeam and Si substrate in (c) and (d) is 43 nm. Dashed lines in (c) and (d) show the interface between SiO_2_ and Ge.

The required voltages to reach relatively high strains (i.e. >2%) on the Ge nanobeam are typically in the range of 100–1000 V for nanobeam lengths in between 200 and 2000 nm as shown in Fig. 3b. Moderate Sn incorporation and n-type doping can be used individually or together to reduce the required strain; and therefore the required voltage, to achieve light emission enhancement. Another method to reduce the required voltage to achieve high strains in a Ge nanobeam is the initial strain induction on the nanobeam. In this study, a 1.5 GPa tensilely-stressed silicon nitride (SiN_x_) layer is assumed to be deposited on Ge nanobeam and patterned as illustrated in Fig. 4a to induce an initial strain. The initial strain introduced in to Ge nanobeam increases with SiN_x_ thickness as shown in Fig. 4b. While only 27 nm-thick SiN_x_ is sufficient to obtain a strain of 1%, 550 nm-thick SiN_x_ is required to obtain a strain of 3% on a Ge nanobeam with length and thickness of 350 nm and 20 nm, respectively. An exemplary initial strain profile on Ge nanobeam is illustrated in Fig. 4c, where a uniform strain of around 2% is achieved by the use of the SiN_x_ stressor. It should be noted that inducing initial strain by tensilely-stressed SiN_x_ deposition slightly deflects the Ge nanobeam upwards (i.e. + z direction) as shown in Fig. 4c. Electrostatic actuation, illustrated in Fig. 1, is utilized to further increase and/or tune the strain in Ge nanobeam (Supplementary Information, Section 4). In the case of an initially-strained Ge nanobeam, the required voltage to reach a final strain of 4% (on the bottom surface of the nanobeam at the symmetry axis) are shown in the inset of Fig. 4b. While 276 V is required to achieve a strain of 4% in a Ge nanobeam with no initial strain (also shown in Fig. 3b), 66 V is sufficient to attain the same strain level when the Ge nanobeam has an initial strain of 3% by the SiN_x_ stressor. Figure 4d shows the axial strain profile for a Ge nanobeam structure having a final strain of 4%, which is initially strained to 2% by the SiN_x_ stressor and 136 V is subsequently applied.

### Possible failure mechanisms of the electrostatic strain induction

One concern which has not been addressed so far is that the electric field intensities on the SiO_2_ layer to achieve relatively high strains mostly exceed the dielectric strength of SiO_2_ (i.e. the maximum value that the SiO_2_ layer can withstand before an electric breakdown occurs), which is around 1.5 V/nm^59^. Among the data presented in Fig. 3, the ones belonging to the structures with high-strain and high-t/L ratio exhibit dielectric breakdown.

**Figure 5:**
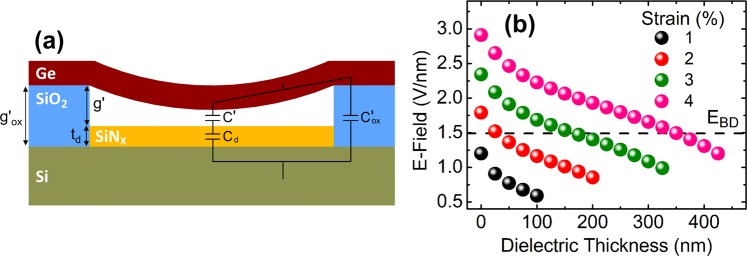
(**a**) Cross-section schematics of the Ge nanobeam structure with SiN_x_ dielectric layer deposited on Si substrate. Capacitors associated with vacuum, slab and SiO_2_ are shown on (**a**). (**b**) The maximum electric field as a function of dielectric layer thickness to achieve 1, 2, 3 and 4% axial strain when L and t are fixed at 350 nm and 20 nm, respectively, and g_eff_ corresponding to each strain is kept constant at 25 nm, 50 nm, 73 nm, 95 nm, respectively. The dashed line indicates the dielectric strength of SiO_2_, E_BD_.

To eliminate dielectric breakdown of SiO_2_ by reducing the electric field intensity on SiO_2_ at a given voltage, one can either increase the dielectric strength of SiO_2_ or increase the gap between the Si substrate and Ge nanobeam. One way to increase the gap without changing the electromechanical force, and therefore the strain on Ge nanobeam, is to insert a dielectric material in between the Ge nanobeam and the Si substrate^60,61^. This dielectric acts as a capacitor serially connected to the capacitor associated with the vacuum layer as shown in Fig. 5a. When the equivalent capacitance of the dielectric-layer and the vacuum-layer capacitances is equal to the vacuum-layer capacitance prior to the dielectric addition, one can achieve the same strain in Ge nanobeam without suffering from the dielectric breakdown as discussed in the Supplementary Information, Section 5, in detail. Therefore, the so-called effective gap distance g_eff_, which is also introduced in the Supplementary Information, Section 5, is adjusted to keep it the same as g of Fig. 1, so that the same axial strain levels are obtained with and without the extra dielectric layer.

The electric field intensity at the SiO_2_ – vacuum interface, where fringing fields are maximum (Supplementary Fig. S8), as a function of dielectric (SiN_x_) thicknesses is shown in Fig. 5b for four different values of axial strain. Electric field intensity developed on SiO_2_ is below its breakdown strength (E_BD_), as shown with the dashed line in Fig. 5b, for an axial strain of 1%. However, it surpasses E_BD_ for higher axial strains and therefore the use of SiN_x_ is required. For example, the thickness of the dielectric layer should exceed 350 nm to obtain 4%-axially-strained Ge nanobeam, where the underlying SiO_2_ does not suffer from dielectric breakdown.

Besides dielectric breakdown, the mechanical fracture of the nanobeam can be considered as another possible failure mechanism. The nanobeam can start fracturing from the highest stress locations when the maximum stress on the nanobeam exceeds the transverse rupture strength. The maximum stress on the nanobeam is calculated to be much lower than the transverse rupture strength for the configuration shown in Fig. 1, and therefore fracture is highly unlikely. However, when a stressed nitride layer is deposited on the nanobeam as depicted in Fig. 4a, the maximum stress on the nanobeam can exceed the threshold value of the transverse rupture strength at the two corners where the nanobeam and nitride meet. Yet, this is not a concern for the initial stress design since the maximum stress can be reduced with fillets, whose formation is unavoidable during microfabrication of nanobeams, as shown in Supplementary Fig. S9 and discussed in Supplementary Information, Section 6.

### Electrical analysis of the actuated Ge nanobeam

**Figure 6:**
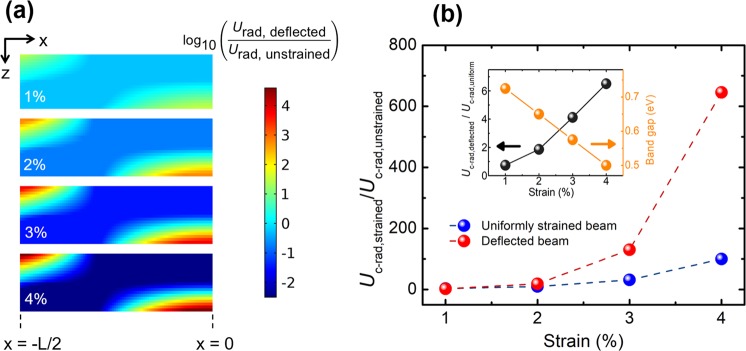
(**a**) Spatial enhancement in *U*_rad_ in one symmetric half of the deflected nanobeam (with L = 200 nm and t = 30 nm) compared to an unstrained nanobeam for strains of 1%, 2%, 3% and 4% under a uniform generation rate of 10^27^ cm^−3^s^−1^. The maximum strain at the two ends of the top surface of the deflected nanobeam is equalized to the one at the bottom surface of the nanobeam at the plane of symmetry by adjusting the curvature of the fillets connecting the Ge nanobeam to SiO_2_ supports as shown in Supplementary Fig. 1. (**b**) The ratio of the cumulative (i.e. integrated over the cross sectional area of the nanobeam) radiative recombination rates in the electromechanically-deflected and uniformly-strained nanobeams to that in the unstrained germanium nanobeam. Inset: The ratio of the cumulative radiative recombination rate of a deflected nanobeam to that of a uniformly strained nanobeam. Dashed lines in (**b**) are to guide the eye.

The non-uniform strain profile in a deflected nanobeam, for example the one demonstrated in Fig. 2a, results in a graded energy bandgap structure (Supplementary Fig. S10). In particular, local minima in bandgap at the bottom portion of the symmetry axis and at the two ends of the top surface of the nanobeam arise since a larger strain occurs in those locations compared to their surroundings. As a result, carriers localize in high concentrations in these highly-strained regions because of the surrounding graded potential barriers^46^. The radiative recombination rate (*U*_rad_) is proportional to the electron-hole product (Equation S18). Therefore, *U*_rad_ at the highly-strained locations in a deflected nanobeam is larger than that at a location in a uniformly-strained nanobeam with a strain that is equal to the maximum strain in the deflected nanobeam. Here, we provided a uniform generation rate in the Ge nanobeam as an input and calculated the resultant *U*_rad_ throughout the nanobeam under steady-state conditions, replicating the photoluminescence experiment, a method commonly used to characterize the light emission efficiency of strained Ge structures^24,27,47^. We compared *U*_rad_ of three structures with the same volume (L = 200 nm, t = 30 nm): (1) the unstrained nanobeam, (2) the uniformly-strained nanobeam, and (3) the electromechanically-deflected nanobeam. The maximum strain of the deflected nanobeam and the strain of the uniformly-strained Ge nanobeam are set to 1%, 2%, 3% and 4% for comparison. The spatial profile of the ratio between the radiative recombination rate of the deflected nanobeam and that of the unstrained nanobeam (*U*_rad,deflected_/*U*_rad,unstrained_) is illustrated in Fig. 6a for various maximum strain values. Furthermore, Fig. 6b shows the ratio of the cumulative radiative recombination rate (*U*_c-rad_) in deflected and uniformly-strained nanobeams to that of an unstrained nanobeam. For a deflected nanobeam with a maximum strain of 1%, *U*_rad,deflected_/*U*_rad,unstrained_ can reach as high as 30 at the location having the highest strain as shown in Fig. 6a. However, due to the smaller *U*_rad_ in the rest of the structure, *U*_c-rad_ of the complete volume of the deflected nanobeam (*U*_c-rad, deflected_) with a maximum strain of 1% is slightly smaller than that in a uniformly 1%-strained nanobeam (*U*_c-rad, uniform_), as shown in the inset of Fig. 6b. The effect of carrier localization becomes more pronounced as the maximum strain increases. For example, the *U*_rad,deflected_/*U*_rad,unstrained_ ratio reaches up to 10^4^ locally for a Ge nanobeam with a maximum strain of 4% as shown in Fig. 6a. This local maximum in *U*_rad,deflected_/*U*_rad,unstrained_ is over two orders of magnitude larger than that achieved in a uniformly-strained Ge nanobeam with the same strain as seen by comparing Fig. 6a,b. Furthermore, *U*_c-rad_ for uniformly-strained and deflected nanobeam at 4% strain is enhanced by around 100 and 600 times, respectively, as compared with the unstrained nanobeam. To sum up, electromechanically-deflected Ge nanobeams are more favorable than their uniformly-strained counterparts in terms of providing enhanced light emission, whose peak emission energy can be tuned between approximately 0.5 eV and 0.75 eV as shown in the inset of Fig. 6b. The tunability of the emission wavelength from 1.65 μm to 2.5 μm is apparent from the calculated PL spectra of the Ge nanobeams for 1, 2, 3, and 4% strains, as shown in Supplementary Fig. S10b.

In summary, we have introduced a Ge nanobeam actuator that allows strain engineering to enhance light emission efficiency as well as wavelength tunability. The results of the FEM simulations provide the required voltage and deflection for obtaining a predefined strain in a given Ge nanobeam. A structure that allows the transfer of the initial strain from the SiN_x_ stressor layer is analyzed, and the results demonstrate that the required voltages can significantly be reduced as compared to the structures without stressor layer. The voltages are expected to be further reduced if moderate Sn incorporation or n-type doping is employed. Two possible failure mechanisms, namely dielectric breakdown and mechanical fracture, can be eliminated by partially filling the region between the nanobeam and the Si substrate with a dielectric slab, and by forming a fillet at the corner of the Ge nanobeam and SiN_x_ stressors, respectively. Finally, we demonstrated that the inevitable graded strain profile on Ge nanobeam brings about a great advantage in terms of light emission efficiency. The actuator introduced in this paper can potentially serve as the key missing component of a monolithically-integrated on-chip IR laser. We anticipate that this work will pave the way for the development of miniaturized systems for biochemical sensing applications and for the demonstration of on-chip optical communications.

## Methods

COMSOL electromechanics module is used to conduct a coupled-field analysis of the structural deformation and the electric field. A triangular mesh with a maximum mesh size of 1/6^th^ of the thickness (E.g. the mesh size in the Ge nanobeam is t/6) is used to guarantee that the results are mesh independent as shown in Supplementary Fig. S11. A Lagrangian-based Green-Lagrange strain tensor together with the second Piola-Kirchhoff stress tensor are used as strain and stress measures, respectively, to compute structural deformation field. The electromechanical model is validated by simulating fixed-fixed edges at small deflection and by comparing the deflection with the analytically-calculated one reported by Choi *et al*.^62^ (Supplementary Fig. S12) as discussed in Supplementary Information, Section 8. As 3D and 2D simulations yield the same results when the width of the nanobeam is larger than 5 times of the thickness (Supplementary Fig. S2), results presented throughout this paper are obtained via 2D simulations to reduce the computation time. One exception is that the simulations regarding initial strain induction via stressor layer are performed in 3D to account for the fact that stressor layer surrounds the Ge nanobeam on the xy plane as schematically illustrated in Fig. 4a.

The fact that the electrostatic pressure can deviate from the normal of the beam is also taken into account. The elasticity of the insulator is also added to the simulation. Si and Ge are taken as infinitely conductive in electromechanical simulations for simplicity, which is reasonable considering the conductivity difference between insulator layers and Ge. The electrical contact is assumed to be located far enough from the nanobeam and therefore has no effect on mechanical simulations. Silicon substrate is assumed to be thick enough such that it does not undergo any deformation. Young’s moduli, densities, Poisson’s ratios and relative permittivities used in FEM simulations are provided in Table S1. Breakdown electric field intensities were calculated by taking the line average of the intensities at the interface of the SiO_2_ layer and the vacuum where the fringing fields are maximum.

Electrical simulations are conducted by Silvaco ATLAS. For these simulations, beam length, beam thickness and fillet radius are taken as 200 nm, 30 nm and 9 nm, respectively, as they give the same maximum axial strain at the edges and at the plane of symmetry, which is crucial for a fair comparison with the results obtained from uniformly strained Ge beam. For computational simplicity, the electromechanically-deflected nanobeam geometry is constructed as planar and rectangular in the electrical domain as shown in Fig. 6a. The axial strain along the curved interior lines (parallel to the bended surfaces) is extracted to form a two-dimensional data set for the strain profile, as if the bended structure is planar. These strain profiles are used to calculate the corresponding narrowing in the bandgap according to the previously conducted experimental studies on uniaxially-strained beams^27^. The calculated band gap profile is provided as an input to the planar Ge nanobeam structure in a material mesh with a resolution of 100 × 20 (100 data points in the x direction and 20 data points in the z direction) with varying bandgap at each pixel. Electronic transitions through the L valley are not considered in the simulations and therefore, the calculations take into account solely the recombinations through the Γ valley as discussed in more detail in Supplementary Information, Section 7. In all of the electrical simulations, Ge is assumed to be undoped with a Shockley-Read-Hall (SRH) lifetime of 5 ns, equal for both electrons and holes. The Auger coefficients for holes and electrons, and the radiative recombination coefficients for the Γ (R_Γ_) and L (R_L_) valleys are provided in Table S1. The changes in Γ and L conduction band minima of Ge, and the resultant change in *n*_Γ_/*n*, which is the ratio of Γ-valley electron concentration to the total electron concentration in conduction band, with increasing strain is assumed in accordance with the experimental and theoretical works conducted on uniaxially-strained samples^11^. The uniform generation rate of 10^27^ cm^−3^s^−1^ is calculated assuming an excitation laser with a spot diameter of 10 μm, laser power of 1 mW, laser wavelength of 532 nm, and a total absorption of 10% of the incident radiation in the 30 nm-thick Ge.

## Supplementary information

Video 1

Supplementary Information
