# Supplementary material for: Strain Engineering of Germanium Nanobeams by Electrostatic Actuation
Source: Sci Rep. 2019 Mar 21;9:4963. doi: 10.1038/s41598-019-41097-1 (PMC6428825; doi:10.1038/s41598-019-41097-1)
Supplement: Supplementary file 2 — Supplementary Information [file 41598_2019_41097_MOESM2_ESM.pdf]

Strain Engineering of Germanium Nanobeams by Electrostatic Actuation

Arman Ayan[1,2,‡], Deniz Turkay[2,3], Buse Unlu[4], Parisa Naghinazhadahmadi[1], Samad Nadimi Bavil Oliaei[5], Cicek Boztug[4], Selcuk Yerci[1,2,3]

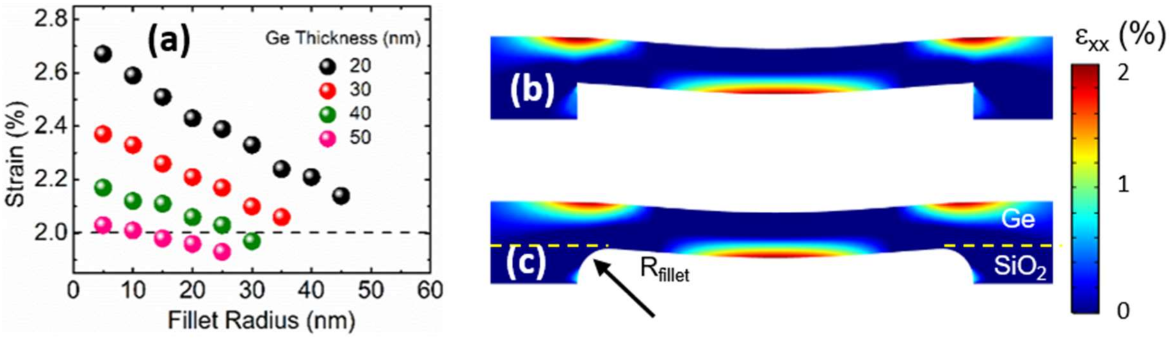

Figure S1. **(a)** The variation of the axial strain at the two edges of the Ge nanobeam's top surface with fillet radius for Ge thicknesses of 20, 30, 40 and 50 nm and a nanobeam length of 350 nm. Axial strain at the symmetry axis is independent from the fillet radius and equal to 2%, indicated by the dashed line in (a). **(b)** Axial strain profile on a Ge nanobeam without a fillet at the Ge nanobeam and SiO_2 corner. **(c)** Axial strain profile on a Ge nanobeam with a fillet radius (R_{fillet}), highlighted by the arrow, of 9 nm. Tensile axial strain at the symmetry plane is equal to 2% in (b) and (c). The length and thickness of Ge nanobeam are 350 nm and 30 nm, respectively, in both (b) and (c). Dashed lines in (c) indicate the interface of Ge and SiO_2. Note that obtaining perfectly sharp corners are practically impossible and the rounded corners, called fillets, typically form during etching of the SiO_2 under the Ge nanobeam.

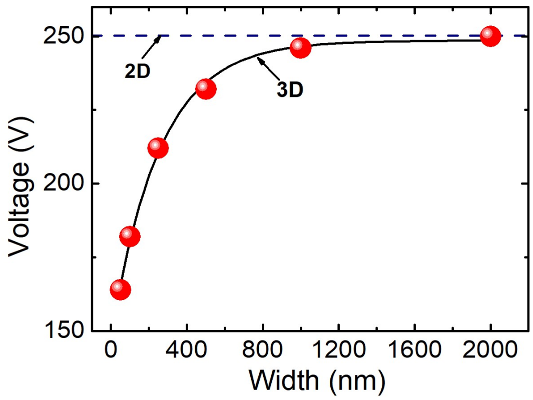

Figure S2. The red symbols indicate the voltage levels required to achieve 2% tensile strain in Ge nanobeam for various Ge nanobeam widths for a fixed thickness and length of 50 nm and 1 μm, respectively. Solid line is the guide to the eye. Dashed line indicates the required voltage to achieve 2% strain calculated using a 2D model.

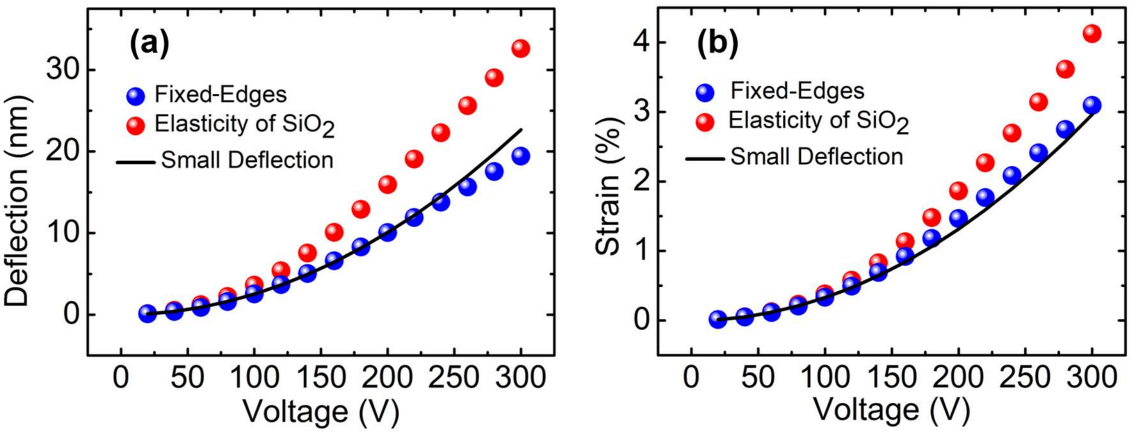

Figure S3. The variation of deflection **(a)** and strain **(b)** with the applied voltage in a Ge nanobeam with the fixed edges and the edges supported by SiO_2. Solid lines in (a) and (b) are values predicted by the small deflection theory summarized in Supplementary Information Section 1. The length and thickness of the nanobeam are 350 nm and 20 nm, respectively. The gap between the Ge nanobeam and Si substrate is 100 nm.

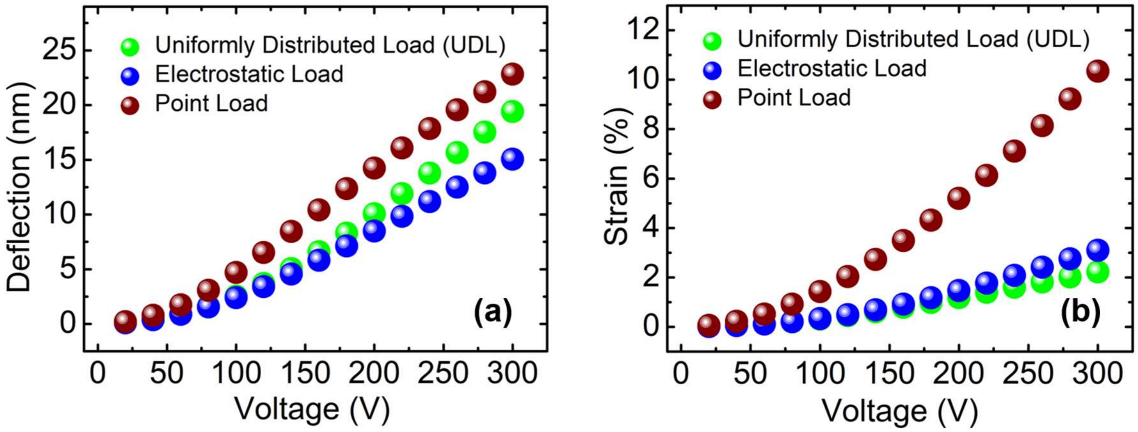

Figure S4. The variation of deflection **(a)** and strain (**b**) with the applied voltage in a Ge nanobeam for a uniformly-distributed load along the Ge nanobeam, a point load at its symmetry axis and a load created by the electrical potential application. The length and thickness of the nanobeam are 350 nm and 20 nm, respectively. The gap between the Ge nanobeam and Si substrate is 100 nm. The integration of uniform load and the electrostatic load over the length of the nanobeam are set to be equal. The magnitude of the point load is equal to the cumulative force in the uniform (or electrostatic) load.

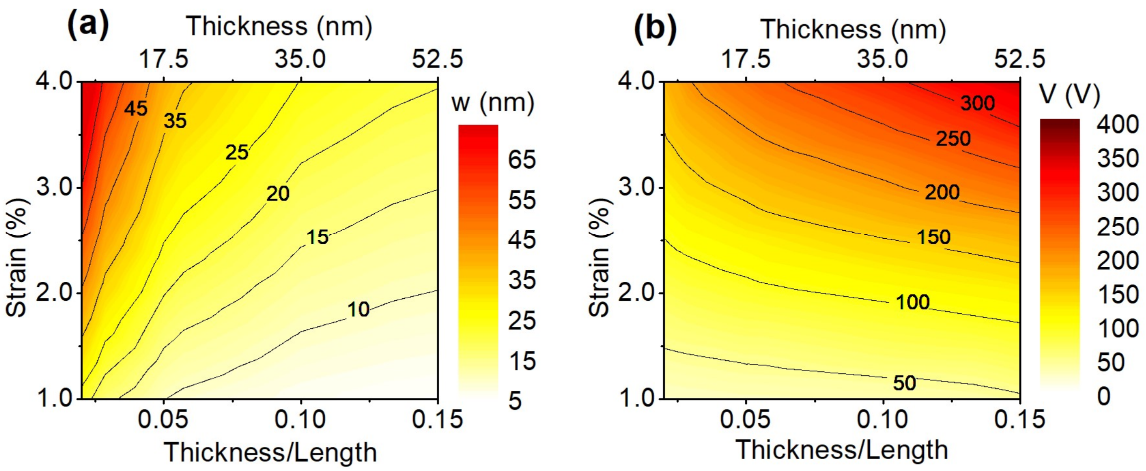

Figure S5. The deflection **(a)** and applied voltage (**b)** at various axial strains and t/L ratios in Ge nanobeams when geometric non-linearities are neglected in the simulations. The distance between the Ge nanobeam and Si substrate is equal to 3 times of the deflection shown in (a)**.** The length of the nanobeam is 350 nm in (a) and (b).

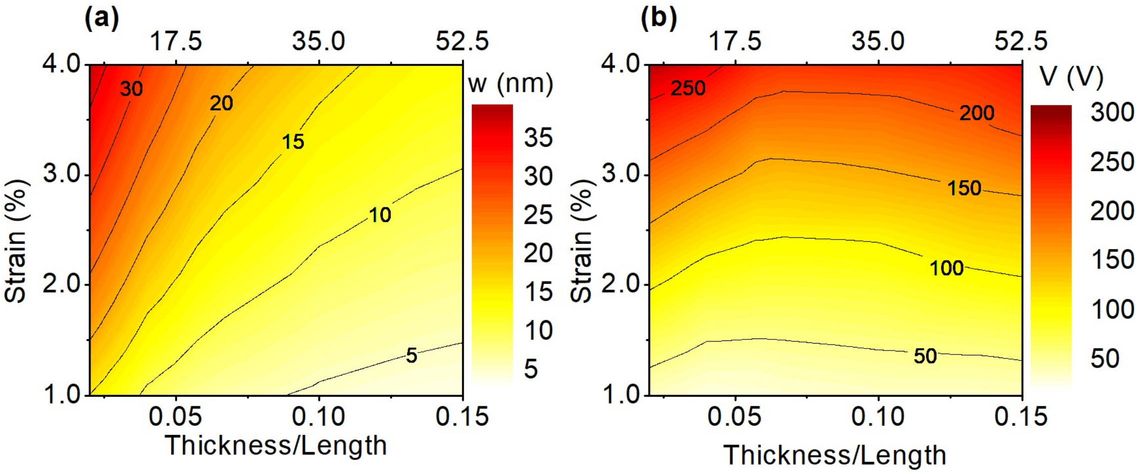

Figure S6. The deflection **(a)** and applied voltage **(b)** at various axial strains and t/L ratios in Ge nanobeams with fixed edges. The distance between the Ge nanobeam and Si substrate is equal to 3 times the deflection shown in (a)**.** The length of the nanobeam is 350 nm in (a) and (b).

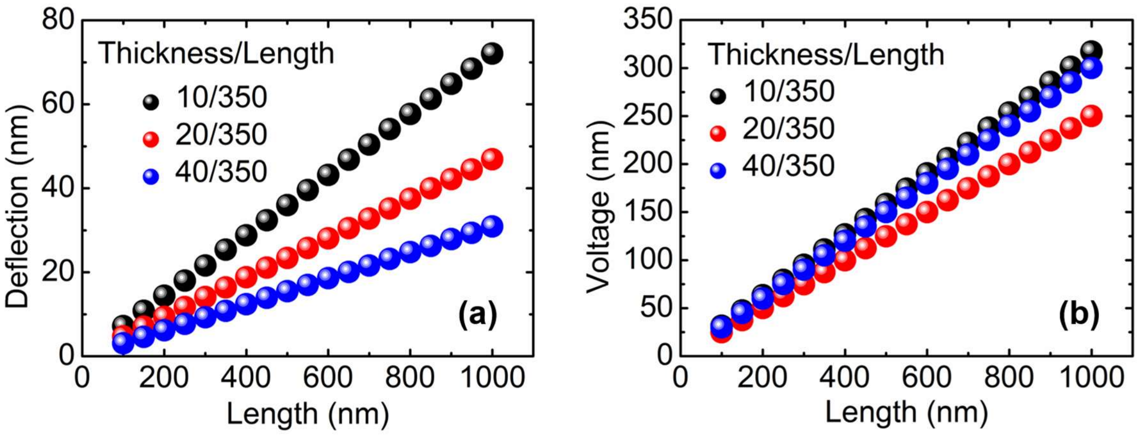

Figure S7. The dependence of the required deflection **(a)** and required voltage **(b)** on Ge nanobeam length for Ge nanobeams with various t/L ratios to achieve 2% tensile strain.

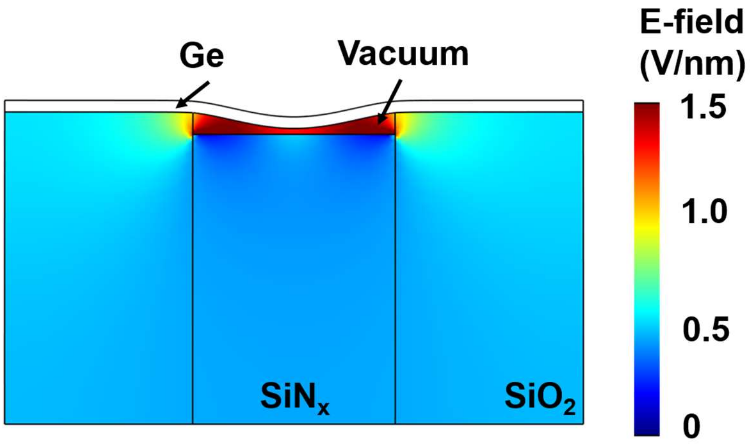

Figure S8. Electric field intensity distribution of the Ge nanobeam structure under 4% strain with a dielectric thickness of 400 nm and an effective gap of 95 nm.

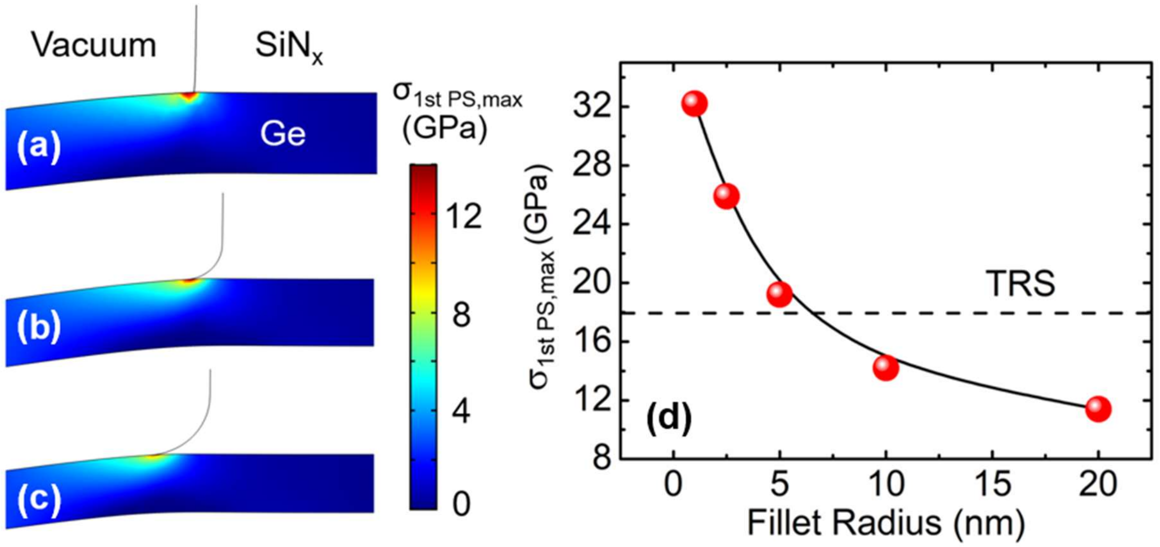

Figure S9. First principal stress distribution on a Ge nanobeam **(a)** in the absence of a fillet, **(b)** with a fillet radius of 10 nm, and **(c)** with a fillet radius of 20 nm at the corner of Ge nanobeam and SiN_x. **(d)** The effect of fillet radius on the maximum first principal stress .The solid line is to guide the eye. The dashed line in (d) shows the transverse rupture strength of Ge.

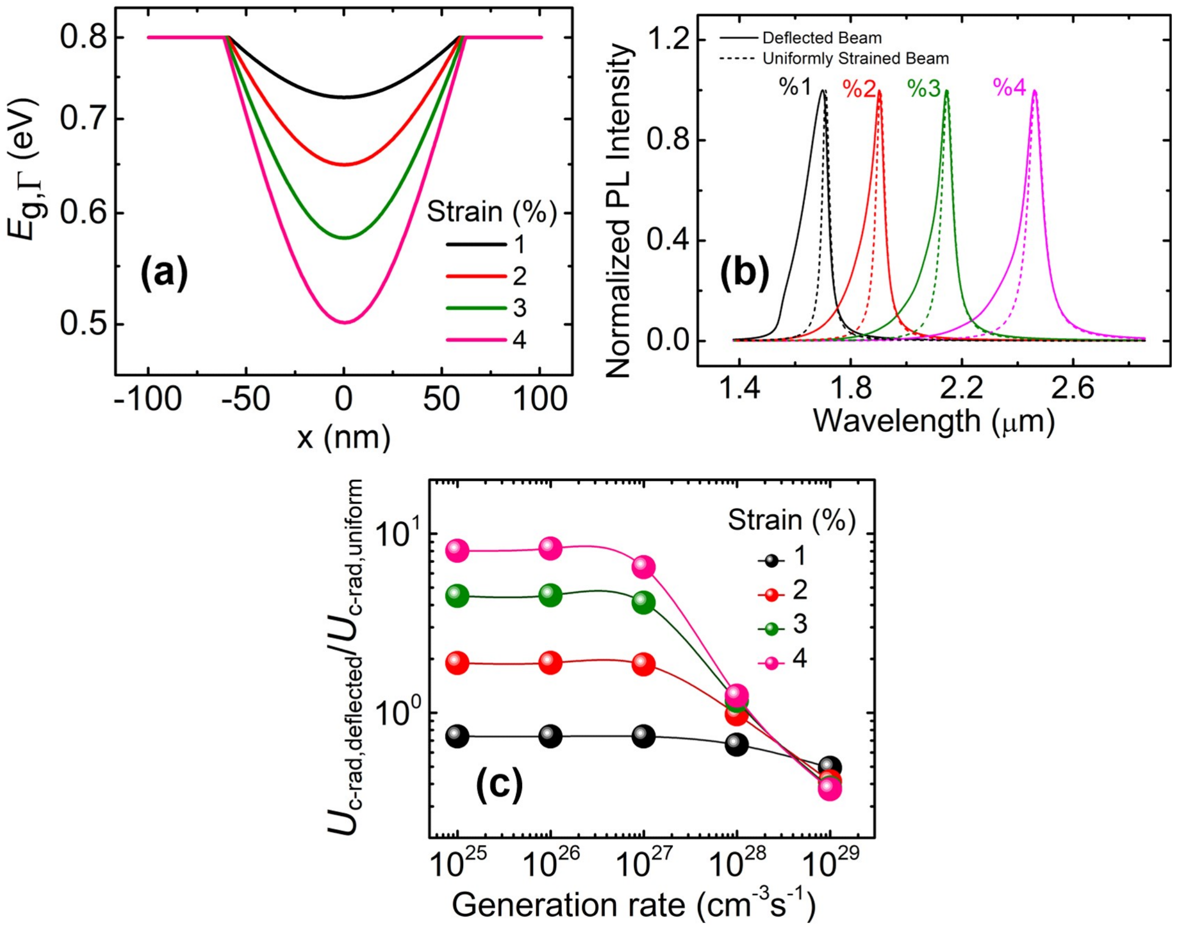

Figure S10. **(a)** The direct band gap profiles at the bottom of the electromechanically-deflected nanobeams for varying maximum strains induced at the symmetry axis. (**b)** The calculated photoluminescence spectra of the deflected and uniformly strained Ge nanobeams for 1%, 2%, 3% and 4% axial strains. **(c)** The ratio of cumulative radiative recombination in a deflected beam to that in a uniformly strained beam as a function of uniform carrier generation rates for various axial strains.

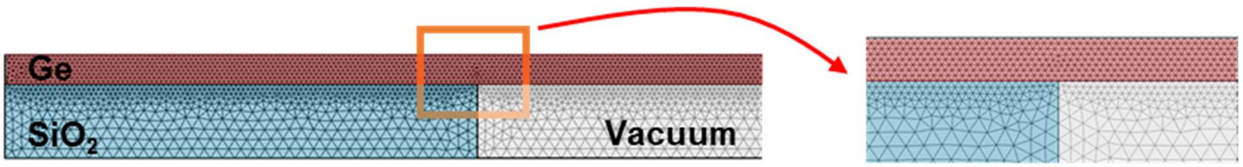

Figure S11. Mesh network of the simulated structure (left). Half of the structure is shown due to the symmetry. A zoomed-in image of the region highlighted by orange is shown on the right.

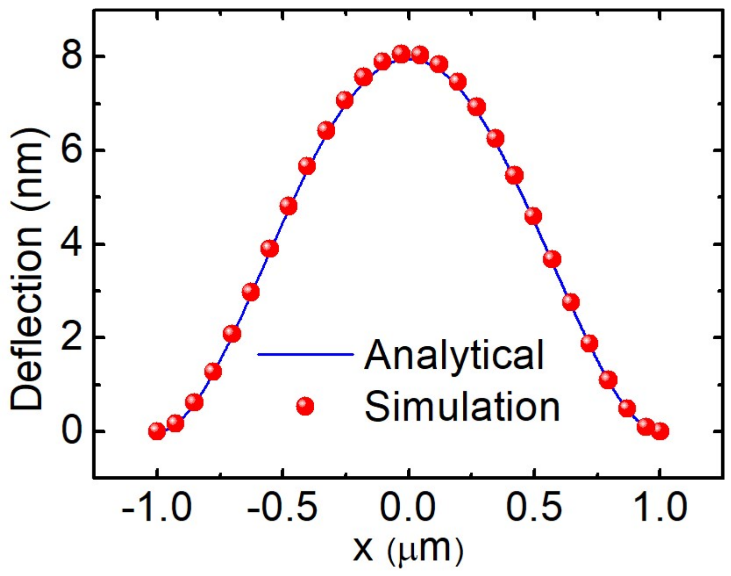

Figure S12. The deflection along the nanobeam simulated by FEM and analytically calculated using Equations (S20) and (S21)[1]. The length and thickness of the Ge nanobeam are 2 μm and 30 nm, respectively. The applied voltage is 10 V. The gap between the Ge nanobeam and Si substrate is 100 nm.

Section 1: Comparison of 2D and 3D Ge nanobeams

The results presented in this paper, except for the ones in the initial strain induction, are obtained via 2D simulations. In a 2D geometry (i.e. when the nanobeam is very wide), electric field forms between the Ge nanobeam and Si substrate as straight lines. However, for relatively narrower nanobeams, the electric field lines between the Si substrate and the top and side surfaces of the nanobeam should be taken into account. These additional electric field lines are called fringing fields. Fringing fields provide an additional force on the Ge nanobeam and reduce the required voltage significantly with decreasing nanobeam width as shown in Figure S2. As a result, this paper presents upper limits of the required voltages for 3D Ge nanobeams.

Section 2: Small Deflection Theory

Analytical solutions obtained under certain approximations are useful to predict the deflection and required voltage, and therefore to confirm the validity of the simulations. Choi et. al[1] presented an

analytical solution for the deflection profile of a deflected beam under an electrical load based on the following assumptions:

1. The deflection is much smaller than the thickness of the nanobeam. Therefore, the geometric nonlinearity effects such as stress stiffening and stretching can be ignored.
2. The force caused by the fringing fields are negligible.
3. The edges are fixed.
4. The deflection is much smaller than the gap distance so that, the electric force does not alter with deflection.

Under the same set of assumptions, a model to estimate the required voltage and the deflection to reach a predetermined strain can be developed. Based on the small deflection theory, the state of uniaxial stress can be assumed, where, around the symmetry axis, upper portions of the beam undergo negative strain and stress, while the lower portions are in tension. This implies that there exists a neutral axis, where strain and stress are zero. Assuming a local curvature 'R_{cv}', the longitudinal strain (ε_{xx}) at a distance of z from the neutral axis can be calculated as:

$$\epsilon_{xx}(x,z) = \frac{z}{R_{cv}} \tag{S1}$$

According to Saint-Venant's principle, Equation (S1) is valid at locations sufficiently far from the supports, where localized distortions are present[2]. Since the nanobeam is prismatic, the neutral axis lie at the mid-plane in the z axis. Thus, the maximum strain at the plane of symmetry can be written as

$$\epsilon_{xx}(x,\frac{t}{2}) = \frac{t}{2R_{cv}} \tag{S2}$$

where t is the thickness of the nanobeam. Since the first derivative of the radius of curvature is zero at the plane of symmetry, the expression becomes

$$\epsilon_{xx}(0,\frac{t}{2}) = \frac{t}{2}\frac{d^2w(x)}{dx^2} \tag{S3}$$

where $w(x)$ denotes the deflection profile of the nanobeam. If the deflection profile is taken as a fourth order polynomial as found by Choi et al.[1],

$$w(x) = w_{max}\left(\left(\frac{x}{0.5L}\right)^2 - 1\right)^2 \quad \text{(S4)}$$

where w_{max} is the maximum deflection and *L* is the length of the nanobeam, then the maximum strain at the plane of symmetry is found as

$$\epsilon_{xx}(0,\frac{t}{2}) = \frac{8t}{L^2}w_{max} \quad \text{(S5)}$$

For the calculation of the maximum deflection, linear stiffness assumption is made. For a fixed-edge prismatic beam, the spring constant under uniform load is

$$k = \frac{32Ebt^3}{L^3} = \frac{F}{w_{max}} \quad \text{(S6)}$$

where *F* is the magnitude of the cumulative electrical force, *E* is the Young's modulus and *b* is the width (breadth) of the nanobeam. The electrical force on a beam can be found by integrating the electrical pressure given in Choi et al.[1] over the electromechanical interface axes, x and y.

$$F = \frac{\epsilon_0 V^2}{2}\int_{-b/2}^{b/2} dy \int_{-L/2}^{L/2} \frac{dx}{g - w(x)^2} \quad \text{(S7)}$$

where ϵ_0 is the permittivity of vacuum, *g* is the gap distance and *V* is the applied voltage. If the deflection dependence of the electrical force is neglected under the small deflection assumption, the electrical force can be found as

$$F = \frac{\epsilon_0 bLV^2}{2g^2} \quad \text{(S8)}$$

Notice that for a better approximation, the deflection profile given in Equation (S4) can be plugged into Equation (S7) and the result would be 1.57 times the force given in Equation (S8). If the Equations (S5), (S6) and (S8) are combined, the maximum deflection and the strain at the symmetry plane can be found as

$$w_{max} = \frac{1}{64}\frac{\epsilon_0}{E}\frac{L^4}{t^3}\frac{V^2}{g^2} \quad \text{(S9)}$$

$$\epsilon_{xx}\left(0,\frac{t}{2}\right) = \frac{1}{8}\frac{\epsilon_0}{E}\frac{L^2}{t^2}\frac{V^2}{g^2} \quad \text{(S10)}$$

When the condition of w_{max} = g/3 is applied to Equations (S9) and (S10) and they are rearranged to provide required voltage and the deflection to reach a predetermined strain value, Equations (S9) and (S10) become

$$w_{max} = \frac{L^2\epsilon_{xx}\left(0,\frac{t}{2}\right)}{8t} \quad \text{(S11)}$$

$$V = \frac{3\sqrt{2}}{4}L\sqrt{\frac{E}{\epsilon_0}}\left(\epsilon_{xx}\left(0,\frac{t}{2}\right)\right)^{1.5} \quad \text{(S12)}$$

Equations (S11) and (S12) predict that the required deflection depends linearly on the maximum strain at the symmetry plane and the required voltage varies with 1.5^{th} power of the maximum strain at the symmetry plane, respectively.

Section 3: Non-linear effects

In Ge nanobeam analysis, since the edges are quasi-fixed, there should be an equivalent axial force to prevent the edges from moving inwards during the deflection. This axial force results in a so-called “nonlinear stretching effect” which, in turn, leads to stress stiffening. Consequently, the stiffness of the nanobeam increases super-linearly with deflection. Thus, the nanobeam undergoes a smaller deflection at an applied voltage compared to what is predicted by the small deflection theory. For nanobeams undergoing sufficiently large deflections, the nonlinear stretching effect at the plane of symmetry can result in large axial tensile strains. These large tensile strains, when superimposed on bending-induced strains (either compressive or tensile), can neutralize compressive strains and can bring about a cross-section with purely tensile strains. While stress stiffening leads to a reduced deflection at a given voltage, strain localization (i.e. extinction of compressively strained regions) enhances strain at the same deflection when

compared to that predicted by the small deflection theory. It is worth mentioning that as t/L ratio increases; the effect of shear deformation on nanobeam deflection becomes more significant, meaning that for large t/L ratios the stiffness of the nanobeam deviates from the relations obtained by disregarding shear deformation.

In addition to geometric nonlinearities, the elasticity of the silicon dioxide on which the Ge nanobeam is resting should be considered. Figure S3 shows that the deflection of and strain on the Ge nanobeam is enhanced when the elasticity of SiO_2 is taken into account under the same applied voltage. Yet the elasticity of SiO_2 results in an increase in the required deflection and voltage to achieve a predetermined strain as can be seen when Figure 3 and Figure S6 are compared. Finally, it should be noted that the distribution of the electrical force on the nanobeam changes as the nanobeam bends. While the electrical force is uniform when there is no deflection, it enhances greatly at the symmetry axis as the nanobeam deflects[1]. As shown in Figure S4 the behavior of deflection under the electrical load resembles that of uniform force for small deflections. As the deflection increases, the force distribution due to electrical load approaches to that of a point load. Similar to the effect of the elasticity of SiO_2, the variation of electrical force distribution with voltage enhances the deflection and strain of the Ge nanobeam with applied voltage as shown in Figure S4. It is worth noting that the model of this study is based on finite element simulations, and it accounts for the effects of non-ideal boundary conditions (i.e. rotations and compressions) at both edges due to the elastic foundation, nonlinear stiffening and the non-uniform electrical force.

Section 4: Initial Strain Induction

When the stressed SiN_x is deposited on the Ge nanobeam, the beam experience a deflection away from the Si substrate, which increases the gap distance. Consequently, when the potential

difference is applied between the Si substrate and the Ge nanobeam, the electrostatic force decreases due to the increased gap distance. Nevertheless, it is possible to decrease the thickness of the SiO_2 layer and achieve the predetermined strain at w=g/3. In Figure 4d, the required voltage values to reach 4% axial strain are presented when

$$w_{final} - w_{initial} = \frac{t_{ox} + w_{initial}}{3} \tag{S13}$$

where w_{final} is the deflection after electrostatic potential is applied, $w_{initial}$ is the deflection due to the nitride stress and t_{ox} is the oxide thickness, which is equal to the gap distance before the stressed SiN_x is deposited.

Section 5: Overcoming the dielectric breakdown of SiO_2

The electrical pressure (i.e. electrical force per unit area) on the Ge nanobeam for the structure shown in Figure 1 can be calculated using Equation (S7) as

$$p = \frac{1}{2}\frac{dC''}{dz}V^2 = \frac{\epsilon_0 \mathrm{V}^2}{2(\mathrm{g}-\mathrm{w})^2} \tag{S14}$$

where C'' is the capacitance per unit area. When a dielectric slab is deposited on the Si substrate (Figure 5a), the electrical pressure becomes

$$p = \frac{1}{2}\frac{dC''}{dz}V^2 = \frac{\epsilon_0 \mathrm{V}^2}{2\left(g' + \frac{\mathrm{t_d}}{\epsilon_\mathrm{d}} - \mathrm{w}\right)^2} \tag{S15}$$

where t_d and ϵ_d are the thickness and the relative permittivity of the dielectric layer, respectively. The pressure terms given by Equation (S14) and Equation (S15) are equal to each other when

$$g' + \frac{t_d}{\epsilon_d} = \mathrm{g} \tag{S16}$$

where $g' + t_d/\epsilon_d$ is defined as the effective gap distance, g_{eff} and g' is the thickness of the vacuum region after deposition of the dielectric layer.

As a result, the SiO_2 layer thickness after the deposition of the dielectric layer, g'_{ox}, becomes equal to the summation of the vacuum and dielectric layer thicknesses, which can be written in terms of g_{eff}, t_d and ϵ_d as follows.

$$g'_{ox} = g' + t_d = g_{eff} + t_d\left(1 - \frac{1}{\epsilon_d}\right) \quad \text{(S17)}$$

It should be noted that the limit of the dielectric thickness, t_{max}, when the deflection is equal to g/3 is equal to $2\epsilon_d g/3$.

Section 6: Fracture Analysis

To predict the possibility of any failure during bending, the transverse rupture strength of Ge[3] (=18 GPa) is set as the failure criterion, where it is assumed that material failure occurs for induced first principal stresses larger than the transverse rupture strength of Ge. In particular, stress concentration due to sharp corners is also considered. This problem is more pronounced when having initial strains as shown in Figure 4, where high stress levels exist at the corners where stressed nitride layer and Ge nanobeam meet. Fillets at the corners effectively reduce the stress levels below the transverse rupture strength of the Ge as shown in Figure S9.

Section 7: Electrical analysis of the actuated Ge nanobeam

The radiative recombination rate (U_{rad}) in the Ge nanobeam is modeled by considering the transitions through the Γ and L valleys only, as[4]

$$U_{radiative} = R_L np + (R_\Gamma - R_L)np\left(\frac{n_\Gamma}{n}\right) \quad \text{(S18)}$$

where $R_\Gamma = 1.3 \times 10^{-10}$ cm^3/s and $R_L = 5.1 \times 10^{-15}$ cm^3/s are the radiative recombination coefficients for the Γ and L valleys[5], n is the total electron concentration in the conduction band, n_Γ is the electron concentration in the Γ-valley, and p is the hole concentration.

For unstrained and undoped Ge, the concentration of the electrons residing in the conduction band of the Γ valley (n_Γ) is much smaller than the total electron concentration in the conduction band and the n_Γ/n ratio is approximately equal to 10^{-4} (0.01%). This ratio increasei.es up to approximately 10^{-2} (1%) for a uniaxial tensile strain of 4%[6]. Furthermore, the radiative recombination through the Γ valley occurs much faster compared to the transitions through L valley with approximately 5 order of magnitude difference between the radiative recombination coefficients ($R_L << R_\Gamma$). As a result, the radiative recombination rate is significantly higher through the Γ valley, specifically at strained locations, and the effect of radiative recombination through the L valley is negligible. Therefore, we implemented a single band structure featuring solely the Γ valley. Since the radiative recombinations through the L valley are ignored, this assumption results in a slight-underestimation of the overall radiative recombination rate for the unstrained structure. Therefore, for both the electromechanically-deflected and the uniformly-strained nanobeams, the calculated U_{rad} enhancements compared to the unstrained structure (i.e. $U_{rad,strained}/U_{rad,unstrained}$) constitute an upper limit of what can practically be achieved. However, also note that since we disregard inter-valley transitions between the Γ and L valleys, this upper limit is likely to be increased when the inter-valley effects are properly accounted for.

Changes in the direct bandgap ($E_{g,\Gamma}$) due to uniaxial strain was calculated following Sukhdeo et al.[7], as given in Equation S19. In this respect, Figure S10a shows the direct bandgap profile at the bottom of the electromechanically deflected nanobeams (at z = t = 30 nm, from x = 0 to x = L = 200 nm) for maximum strain values varying between 1% to 4%.

$$E_{g,\Gamma}(eV) = 0.8 - 0.075\ x\ \varepsilon\ (\%) \tag{S19}$$

where ε is the strain in percents.

In the calculation of the photoluminescence (PL) spectra, the radiative recombination rate from each mesh region is retrieved from SILVACO. We took into account the tensilely strained portions of the nanobeam only, and assumed that the emission from each tensilely strained mesh region is due to the electron-light hole radiative recombination with a Lorentzian spectral line shape. The Full-Width Half Maximum (FWHM) of the Lorentzian function is taken as $2\hbar/\tau$ to account for the lifetime broadening where τ is the intraband scattering lifetime, which has a typical value of 100 fs[7]. A similar approach has been previously used to determine the theoretical spontaneous emission spectra of the strained Ge nanomembranes and the results are found in agreement with the experimental results[8]

The normalized PL spectra of the deflected and uniformly strained Ge nanobeams with 1, 2, 3, and 4% tensile strains are shown in Figure S10b. The FWHM values of the PL spectra of the deflected Ge nanobeams are significantly larger than that of uniformly strained Ge nanobeams at small strains due to non-uniform bandgap profile of the structure. However, carrier localization to the highest strain locations at the deflected Ge nanobeams become more pronounced as the strain increases. Therefore, the FWHM values of the PL spectra of the deflected Ge nanobeams approach to that of uniformly strained Ge nanobeams.

The illumination intensity can be varied in the photoluminescence measurements, and depending on the optical design of the nanobeam structure, the generation rate in the structure can vary. Moreover, the total generation rate constitutes transitions from the valence band to both the Γ and L valleys. Thus, the generation rate to the Γ valley is less than the total generation rate. Due to these factors, the calculated generation rate (*G)* of 10^{27} $cm^{-3}s^{-1}$ (which was calculated assuming that the generation occurs solely between the valence band and Γ valley) is actually subject to changes depending on the strain profile and the optical setup. Therefore, the electrical response

of the nanobeams to varying G is different for the electromechanically deflected and the uniformly strained nanobeams. Auger recombination plays a significant role in the presence of elevated carrier concentrations. Due to the carrier localization mechanism in the deflected nanobeams, Auger recombination can become a dominant factor on total recombination rate. Thus, the advantage of carrier localization in the deflected nanobeams can be shadowed by the elevated Auger recombination rates, and turn to a disadvantage under high illumination levels. In this respect, Figure S10c shows the changes $U_{\text{c-rad,deflected}}/U_{\text{c-rad,uniform}}$ under varying G. Under low to moderate illumination levels ($G < 10^{27}$ cm^{-3}s^{-1}) and high maximum strains (>%1), the deflected beams are advantageous compared to the uniformly strained structures in terms of $U_{\text{c-rad}}$. On the other hand, for high illumination levels (G > 10^{28} cm^{-3}s^{-1}), the electromechanically deflected nanobeams can actually become disadvantageous since the carrier localization is not beneficial anymore.

In the electrical simulations, 200 nm of beam length, 30 nm of beam thickness and 9 nm fillet radius are chosen as they give the same maximum axial strain at the edges and at the plane of symmetry. This is because, obtaining the same strain is crucial for a fair comparison with the uniform strain case.

Section 8 : Validity of Simulations

To check the reliability of the simulations, the analytical equations given in the study of the Choi et. al.[1] are regenerated. In that paper, the beam which is fixed at the two edges is analyzed. In their study, when deflection is significantly lower than the gap, the deflection profile is found as

$$w(x) = \frac{\epsilon_0 b V^2}{48\,EIg^2}(x^2 - l^2)^2 \tag{S20}$$

where

$$I = \frac{bt^3}{12} \quad \text{(S21)}$$

where ϵ_0 is the permittivity of the vacuum, V is the applied potential between the two terminals separated by the gap, b is the width of the beam, E is the Young Modulus of the material, I is the moment of inertia, g is the gap, t is the thickness of the beam and l=L/2 is the half of the length of the beam. For the L = 2 μm, g = 100nm, t = 30 nm V = 10 V and E=103 GPa, the fixed-fixed case is simulated and the results are in a good agreement with the analytic solutions as it is shown in Figure S12.

Table S1. The Young's moduli, Poisson's ratios, densities of Si and SiO_2, relative permittivities of SiO_2 and Si_3N_4, radiative recombination coefficients at Γ and L valleys, Auger recombination coefficients of electrons and holes, Shockley-Read-Hall lifetime (assumed to be equal for both electrons and holes) of Ge and the operation temperature of the system.

Parameters	Value (Unit)	Parameters	Value (Unit)
E_{Ge}	103 GPa	R_{Γ}	1.3 x 10^{-10} cm^3/s
E_{SiO2}	70 GPa	R_L	5.1 x 10^{-15} cm^3/s
v_{Ge}	0.26	C_{ppn}	7x10^{-32} cm^6/s
v_{SiO2}	0.17	C_{nnp}	3x10^{-32} cm^6/s
d_{Ge}	5323 kg/m^3	τ_{SRH}	5 ns
d_{SiO2}	2200 kg/m^3	T	300 K
ϵ_{SiO2}	3.9		
ϵ_{SiNx}	7.5		

References

1. Choi, B. and Lovell, E. G. Improved analysis of microbeams under mechanical and electrostatic loads. *J. Micromechanics Microengineering* **7,** 24 (1997).
2. Popov, E. & Balan, T. *Engineering mechanics of solids*. 467-468 (1990).
3. Smith, D. A., Holmberg, V. C. & Korgel, B. A. Flexible Germanium Nanowires: Ideal Strength, Room Temperature Plasticity, and Bendable Semiconductor Fabric. *ACS Nano* **4,** 2356–2362 (2010).
4. Sukhdeo, D. S., Gupta, S., Saraswat, K. C., Dutt, B. (Raj) & Nam, D. Impact of minority carrier lifetime on the performance of strained germanium light sources. *Opt. Commun.* **364,** 233–237 (2016).
5. Liu, J. *et al.* Tensile-strained, n-type Ge as a gain medium for monolithic laser integration on Si. *Opt. Express* **15,** 11272–11277 (2007).
6. Sukhdeo, D. S., Nam, D., Kang, J.-H., Brongersma, M. L. & Saraswat, K. C. Direct bandgap germanium-on-silicon inferred from 5.7% <100>; uniaxial tensile strain [Invited]. *Photonics Res.* **2,** A8–A13 (2014).
7. Coldren, L. A., Corzine, S. W. & Mashanovitch, M. L. *Diode lasers and photonic integrated circuits*. **218,** 132 (John Wiley & Sons, 2012).
8. Sánchez-Pérez, J. R. *et al.* Direct-bandgap light-emitting germanium in tensilely strained nanomembranes. *Proc. Natl. Acad. Sci. U. S. A.* **108,** 18893–8 (2011).
